# Variation in Root-Related Traits Is Associated With Water Uptake in *Lagenaria siceraria* Genotypes Under Water-Deficit Conditions

**DOI:** 10.3389/fpls.2022.897256

**Published:** 2022-06-02

**Authors:** Rodrigo Iván Contreras-Soto, Dinoclaudio Zacarias Rafael, Leonel Domingos Moiana, Carlos Maldonado, Freddy Mora-Poblete

**Affiliations:** ^1^Instituto de Ciencias Agroalimentarias, Animales y Ambientales, Universidad de O' Higgins, San Fernando, Chile; ^2^Agricultural Research Institute of Mozambique, Maputo, Mozambique; ^3^Institute of Biological Sciences, University of Talca, Talca, Chile

**Keywords:** bottle gourd, phenotypic plasticity, root system, water-deficit, water productivity

## Abstract

In many agricultural areas, crop production has decreased due to a lack of water availability, which is having a negative impact on sustainability and putting food security at risk. In plants, the plasticity of the root system architecture (RSA) is considered to be a key trait driving the modification of the growth and structure of roots in response to water deficits. The purpose of this study was to examine the plasticity of the RSA traits (mean root diameter, MRD; root volume, RV; root length, RL; and root surface area, SA) associated with drought tolerance in eight *Lagenaria siceraria* (Mol. Standl) genotypes, representing three different geographical origins: South Africa (BG-58, BG-78, and GC), Asia (Philippines and South Korea), and Chile (Illapel, Chepica, and Osorno). The RSA changes were evaluated at four substrate depths (from 0 to 40 cm). Bottle gourd genotypes were grown in 20 L capacity pots under two contrasting levels of irrigation (well-watered and water-deficit conditions). The results showed that the water productivity (WP) had a significant effect on plasticity values, with the Chilean accessions having the highest values. Furthermore, Illapel and Chepica genotypes presented the highest WP, MRD, and RV values under water-deficit conditions, in which MRD and RV were significant in the deeper layers (20–30 and 30–40 cm). Biplot analysis showed that the Illapel and Chepica genotypes presented a high WP, MRD, and RV, which confirmed that these may be promising drought-tolerant genotypes. Consequently, increased root diameter and volume in bottle gourd may constitute a response to a water deficit. The RSA traits studied here can be used as selection criteria in bottle gourd breeding programs under water-deficit conditions.

## Introduction

Drought is a compelling ecological issue that significantly damages plant development and growth (Fahad et al., 2017; Iqbal et al., 2022). In this sense, water scarcity has had a negative impact on crop yield, putting food security at risk in many drought-prone areas in the world (Ellsworth et al., 2020; Payus et al., 2020; Fahad et al., 2021a,b,c). Therefore, in order to enhance the sustainability of agriculture, it is necessary to develop genotypes with high yields, water-use efficiency, and water productivity (WP). In response to drought stresses, plants have developed several physiological, phenological, morphological, and biochemical adaptation mechanisms in different plant organs (i.e., roots, stems, and leaves) to respond to severe water stress (Fahad et al., 2017, 2021c,d,e,f; Mashilo et al., 2018; Kamran et al., 2019; Zacarias Rafael et al., 2020; Iqbal et al., 2022). In particular, root system architecture (RSA) plays an important role in water extraction from the soil, especially in drought environments (Strock et al., 2019; Fahad et al., 2021b). Therefore, the identification and improvement of genotypes based on root traits have been of interest in crop breeding, mainly for the maintenance of crop productivity under water-deficit conditions (Lynch, 2018; Iqbal et al., 2022). Root system architecture traits are important drivers of many ecosystem processes, such as carbon and nutrient cycling, as well as the response to water-deficit episodes (Lynch, 2018; Lozano et al., 2020). However, the responses of RSA traits in plants under drought conditions have been limited to a small number of plant species (Lozano et al., 2020). In fact, the conclusions about plant strategies in terms of root trait responses have been contradictory. Henry et al. (2012), for instance, concluded that rice plants with fine roots that have smaller root diameters and specific root lengths are better adapted to dry conditions, as they allow the conservation of water resources. Similarly, Awad et al. (2018) observed that smaller root diameters in winter wheat plants may be beneficial for mining water under drought conditions since they may have faster root growth and may be able to allocate more C to root length. This relationship between root length and diameter in plants under drought conditions was also addressed by Comas et al. (2013) and Wasaya et al. (2018). In contrast, Zhou et al. (2018), through a meta-analysis study, found that root length and root length density decreased significantly in response to drought, while root diameter increased. In this sense, Lozano et al. (2020) pointed out that an increase in root diameter and a reduction in root elongation are different strategies that may promote nutrient and water acquisition, depending on the plant species. Thus, root system traits can vary from plant to plant, and also from species to species in response to the environment.

The ability of plant roots to capture water and nutrients from different depths in the soil profile is related to phenotypic variation and plasticity in root system traits, such as root length, diameter, area, and volume (Gorim and Vandenberg, 2017; Brunel-Saldias et al., 2020). For example, Gorim and Vandenberg (2017) pointed out that, between depths of 40–60 cm, lentil genotypes with higher total root length (TRL) proportions of very fine roots (<0.5 mm) were able to exploit both water and nutrients more efficiently; while between depths of 0–20 and 20–40 cm, lentil genotypes with TRL diameters >2.0 mm were more efficient in the long-distance transportation of water and nutrients. Similarly, Brunel-Saldias et al. (2020) showed that, under a water-limited regime, water use (WU) in spring wheat was positively related to root weight density at different soil layers. Thus, while root traits such as length, diameter, area, and volume are important, more information about root systems can be gained by looking at root distribution patterns at various soil depths.

Bottle gourd [*Lagenaria siceraria* (Mol. Standl)] was one of the earliest plant species domesticated for human utilization (Decker-Walters et al., 2004). According to Kistler et al. (2014), this species originated in Africa and was taken from Africa to Eurasia by humans, but it reached America by natural transoceanic dispersal. Due to this dispersal, *L. siceraria* is divided into two subspecies: Asian bottle gourd [*L. siceraria*. subsp. *asiatica* (Kobyakova) Heiser] and American/African bottle gourd (*L. siceraria*. subsp. *siceraria*) (Kobiakova, 1930; Schlumbaum and Vandorpe, 2012). Currently, bottle gourd is widely used as a rootstock in watermelon due to its tolerance to different biotic and abiotic stress factors (Yetisir et al., 2003; Yetisir and Uygur, 2009; Ulas et al., 2019; Aslam et al., 2020; Yavuz et al., 2020). Notably, bottle gourd has been an important crop in the arid and semi-arid regions of sub-Saharan Africa, where heat and drought stresses are major constraints for crop production (Sithole and Modi, 2015; Mashilo et al., 2017). Landraces or varieties of bottle gourd from Africa are thought to be drought tolerant due to several years of selection and cultivation by farmers living in drought-prone areas (Mashilo et al., 2017). Thus, these materials may possess unique genetic, physiological, and morphological attributes that may not be present in modern varieties, making them potentially key genetic resources for crop improvement (Zacarias Rafael et al., 2020).

A recent study conducted with bottle gourd reported that drought-tolerant genotypes showed a reduced length and density of lateral roots, constituting a response to a water deficit (Zacarias Rafael et al., 2020). However, until now, no study has examined the length, diameter, surface area, and volume root patterns at different substrate depths in the *L. siceraria* species under drought conditions. Therefore, in this study, eight bottle gourd genotypes, representing three different geographical origins (i.e., South Africa, Asia, and Chile), were selected to determine changes in the root system at different substrate depths under drought and well-watered conditions. This information will be useful for the development of bottle gourd varieties that can tolerate drought stress conditions by developing optimal root systems.

## Materials and Methods

### Plant Material

The plant material used in this study consisted of eight bottle gourd accessions that represented different geographical origins: South Africa, Asia, and Chile. Three accessions were sourced from the Limpopo Department of Agriculture and Rural Development (Towoomba Research Station) of South Africa, two accessions were from the Genetic Resource Center of Japan, specifically from the National Agriculture and Food Research Organization (NARO), and three were collected from three different regions of Chile. Details about the bottle gourd accessions are shown in Supplementary Table 1.

### Experimental Design and Growing Conditions

The seeds of the bottle gourd accessions were sterilized by immersion in 2% (v/v) sodium hypochlorite in water for 10 min, rinsed 2 times with deionized water for 10 min, and germinated for 5–7 days at 20–25°C in 7 × 7 × 8 cm (0.23 L) pots with peat and sand substrate in an equal ratio of 1:1. The plants with the first fully expanded true leaf and with an absence of damage or disease were considered as criteria for transplantation to pots.

The experiment was conducted in February 2021 in glasshouse conditions using a shade net cover (Raschel sun-shading net with 50% light transmittance), the average air temperature was 23.8 ± 2.7°C with relative humidity of 54% and a solar radiation level of 27 Mj/m^2^. The experiment was established in a randomized complete block design (RCBD) with an 8 × 2 factorial arrangement and three replicates by block. Factors consisted of eight bottle gourd accessions and two water regimes (well-watered and water-deficit regimes) totaling 16 treatments.

Each pot with 20 L of capacity (top diameter 30 cm, bottom diameter 26.5 cm, and height 40 cm) was filled with 30 kg of the substrate (1:1 peat/sand v/v) (volume 0.01766 m^3^). Prior to transplant, each pot was saturated with water, allowed to drain, and covered with plastic bags to avoid evaporation for 24 h according to Opazo et al. (2020). Thereafter, the weight of each pot was recorded and this was established as 100% of substrate water content and considered as field capacity. Three days after transplanting, the plants were subjected to two irrigation conditions: well-watered (WW) and water-deficit (WD). Plants under the WW condition were irrigated 3 times per week during the period of the experiment (35 days), with water being added to each pot to reach the corresponding 100% of field capacity. In contrast, the WD condition was induced by suspending the irrigation supply for 35 days. All pots in WW and WD were weighed 3 times per week to determine the amount of water consumed by each assessed genotype. All pots included holes for water drainage. The plants were fertilized individually with 5 g of Basacote^®^ Mini 3M 16-8-12(+2) N-P_2_O_5_-K_2_O(+MgO+S) at the initial stage of the experiment, after which no fertilizer was applied during the entire experiment to avoid confusion about the applied stress. The experiment ended when the weight of the pots under WD conditions had no variations and the plants had died.

### Water Productivity

Water productivity (WP) was estimated from the water-supplied data. To avoid evaporation, the surface of the pot was covered with black bags. For that, three plants per genotype and water treatment were harvested at the end of the experiment. Root and aerial (leaves and shoots) tissue dry matter weights (RDW and SDW, respectively) were measured using the leaves, shoots, and roots of each plant, which were separated and dried in an oven at 60°C to obtain the dry weights. The root:shoot ratio was determined by dividing the RDW and SDW for each plant. The plant water consumed over the 4-week period was estimated from the sum of the daily water consumption (WU) minus the water drained out of the pots and plant biomass gain. For this, the water drained was estimated through control pots, which included holes for water drainage (without plants), while the plant biomass gain (PBG) was estimated at the end of the experiment and corresponds to PBG = [final fresh weight—initial fresh weight]/[number of days in the experiment]. The WU was determined as follows:


$$\begin{array}{l} WU\;=\;\overset{n}{\underset{i}{\sum }}\left(PW_{i\;-\;1}\;-\;PW_{i}\right)\;-\;\left(CPW_{i\;-\;1}\;-\;CPW_{i}\right)\;-\;PBG_{i} \end{array}$$

where *i* corresponds to each of the *n* measurements (being *i*-1 the past measurement), PW and CPW represent the weight of the pots and control pot, respectively. Finally, the WP was determined as follows:


$$\begin{array}{l} WP\left(kg\;m^{-3}\right)\;=\;\frac{\left(RDW\;+\;SWD\right)}{WU} \end{array}$$

### Root Measurements

The whole root system of each of the bottle gourd plants was analyzed. The root length (RL), root surface area (SA), mean root diameter (MRD), and root volume (RV) were determined using WinRhizo 2019a software (Regent Instruments Inc., Canada). The roots were cleaned by washing them over a sieve. Each sample was scanned with a flatbed scanner (Epson Perfection V800 Photo and V850 Pro, SEIKO EPSON CORP., Japan, resolution 6400 dpi). The roots were partitioned into nine diameter classes in 0.5 mm steps (0.0–0.5, 0.5–1.0, 1.0–1.5, 1.5–2.0, 2.0–2.5, 2.5–3.0, 3.0–3.5, 3.5–4.0, and 4.0–4.5), and the root lengths for each root diameter class were computed. The root images were analyzed at four different substrate depths (0–10, 10–20, 20–30, and 30–40 cm).

### Morphological Plasticity Index

The phenotypic plasticity for a trait is related to the difference in this trait between two individuals of the same genotype under different water conditions (Marchiori et al., 2017). Therefore, the phenotypic plasticity was described by the absolute distance between two selected individuals (j and j', j = 1, 2, and 3) of the same genotype grown under distinct water conditions (i and i', i = WW and WD). According to the above, for a given trait x, the distance among values (dij → i′j′) is the difference xi′j′ – xij, and the relative distances (rdij → i′j′) are defined as dij → i′j′/(xi′j′ + xij) for all pairs of individuals of a given variety grown under different water availability. Finally, the relative distance plasticity index (RDPI) was calculated as Σ(rdij → i′j′)/n, where n represents the number of distances.

### Statistical Data Analysis

An analysis of variance (ANOVA) was performed after testing the homogeneity of variances and the normality of the residuals using Bartlett and Shapiro–Wilk tests, respectively. A two-way ANOVA was performed for RSA traits, WU and WP traits, while a one-way ANOVA was used for RDPI traits (WP, root:shoot, RDW, SDW, RL, SA, RV, and MRD). Fisher's least significant difference (LSD) test and orthogonal contrasts were performed on multiple comparisons of the mean values of the genotypes. Statistical analyses were carried out using R 4.0.5 software (R Core Development Team, 2020).

The mean values of the studied RSA, WU and WP traits for each condition (WW and WD) were used to compute the Pearson's linear correlation coefficients using the “chart. Correlation” function of the PerformanceAnalytics package (Peterson et al., 2018) in R 4.0.5 software (R Core Development Team, 2020).

A principal component analysis (PCA) based on the correlation matrix was performed using the “princomp” function in R. The PCA-biplot was then generated using the “ggbiplot” function of ggbiplot2 package (Vu, 2011) in R to describe and group bottle gourds according to their level of drought tolerance pursuant to Zacarias Rafael et al. (2020).

## Results

### Analysis of Variance and Correlations Among WP, Biomass Production, and Root Traits

The root dry weight (RDW) of the plants in the drought treatment ranged from 1.2 (Osorno) to 2.6 g (GC); while for the well-watered (WW) plants, the dry weight varied from 2.0 (Philippines) to 4.6 g (GC). The leaves and shoots dry matter weight (SDW) ranged from 14.4 (Philippines) to 22.4 g (Chepica) for plants under water-deficit (WD) conditions and 68.8 (Philippines) to 119.7 g (GC) for WW (Supplementary Table 2). There was a significant difference (*p* < 0.01) in the RDW among two South African bottle gourd genotypes (BG-78 and GC) and the Osorno, Philippines, and South Korea genotypes in the drought treatment, which was not observed in WW (Supplementary Table 2). This indicates that, in WD conditions, the South African genotypes have higher root growth compared with the Osorno and Asiatic bottle gourd. It should be noted that, under this same condition, the Osorno genotype had the lowest water use (WU), which coincides with its root growth (Supplementary Table 2).

Analysis of variance showed a significant effect of the water regime in all traits except for WP (Table 1). Moreover, genotypic significant differences were observed under root:shoot ratio (root:shoot), RDW, root length (RL), and root surface area (SA) (Table 1). Similarly, the genotypic effects were significant for root:shoot and RL under the WW condition, while under the water-deficit condition, RDW and RD were significant (Supplementary Table 2). The South African genotypes exhibited the highest values of biomass production (RDW and SDW) and RL, while two of the Chilean genotypes (Illapel and Chepica) showed the highest values in mean root diameter (MRD), root volume (RV), and SA. In particular, in the WD regime, the Illapel and Chepica genotypes showed MRD values that were significantly higher than those of the Asiatic and South African bottle gourds; while for RDW, the BG-78 and GC genotypes displayed significantly greater values than those of the Asiatic bottle gourds (Supplementary Table 2).

**Table 1:** ANOVA results for the effect of water regime (W), genotype (G) and their interaction (G^*^W) on water consumption (WU), water productivity (WP), root and shoot biomass (RDW and SDW, respectively), and root traits (RL, Root length; MRD, Mean Root Diameter; RV, Root Volume; SA, Surface Area) evaluated in eight bottle gourd genotypes under well-watered (WW) and water-deficit (WD) conditions.

**SV**	**WU**	**WP**	**Root:shoot**	**SDW**	**RDW**	**RL**	**MRD**	**RV**	**SA**
G	ns	ns	*	ns	*	***	ns	ns	*
W	***	ns	***	***	**	***	*	**	***
G*W	ns	ns	ns	ns	ns	ns	ns	ns	ns
BG-58	15.44a	4.41a	0.07bcd	68.76a	3.03ab	781.12ab	2.14a	29.07a	526.37ab
BG-78	15.04a	4.16a	0.08abcd	62.46a	3.22a	700.9abc	2.5a	35.61a	548.06ab
GC	15.82a	4.10a	0.1a	67.79a	3.59a	813.74a	2.21a	32.69a	564.94ab
Philippines	11.33a	3.61a	0.09abc	41.60a	1.94bc	449.07e	2.19a	18.26a	313.72c
South Korea	12.73a	4.50a	0.06cd	56.27a	2.14abc	564.97cde	2.1a	20.92a	382.6bc
Illapel	12.60a	3.93a	0.09ab	46.53a	3.17ab	626.52bcd	3.09a	51.25a	610.92a
Chepica	11.96a	4.19a	0.07bcd	47.41a	2.57abc	591.82cde	3.02a	48.97a	568.88ab
Osorno	13.98a	4.30a	0.06d	66.86a	1.77c	500.99de	2.3a	24.72a	382.91bc
*WW*	*21.85*	*4.36*	*0.04*	*95.62*	*3.31*	*698.09*	*2.68*	*43.15*	*591.93*
*WD*	*5.37*	*3.94*	*0.12*	*18.88*	*2.10*	*543.30*	*2.24*	*23.25*	*391.77*

SV, source of variation. The levels of significance (ns, non-significant; *significant at 5%; **significant at 1%; ***significant at 0.1% using the F-test) are indicated. Means with different letters within a column are significantly different at p < 0.01 according to the Fisher's LSD test. ns,

*, **, *** and letters are defined in the table foot.

In most of the tested bottle gourd genotypes, the contrasting means in the comparison of the WW and WD water regimes (Table 2) showed that, for WU and SDW, drought stress significantly reduces their values, while root:shoot significantly increased its values under this watering regime. In general, the water-deficit treatment led to a reduction in all traits, except in some genotypes (WP and root:shoot). Notably, the Illapel and Chepica genotypes showed the highest values of WP under the WD condition (being even higher than those of the WW regime), while the GC and Philippines bottle gourds showed the lowest values of WP (Supplementary Table 2).

**Table 2:** Orthogonal contrasting test for the difference of mean values between water-deficit (WD) and well-watered (WW) conditions for the water consumption (WU), water productivity (WP), root and shoot biomass (RDW and SDW, respectively), and root traits (RL, Root length; MRD, Mean Root Diameter; RV; Root Volume; SA, Surface Area) evaluated in eight bottle gourd genotypes.

**Genotypes**	**WU**	**WP**	**Root:shoot**	**RDW**	**SDW**	**RV**	**RL**	**SA**	**MRD**
BG-58	19.2*	1.07 ns	−0.08**	1.58*	96.7*	19.4*	299.3 ns	275.4*	0.41 ns
BG-78	18.65*	0.42 ns	−0.09*	1.38 ns	84.8 ns	21.0 ns	41.9 ns	173.7 ns	0.57 ns
GC	21.14**	1.11 ns	−0.13*	2.06 ns	103.8*	29.9*	422.5*	404.1**	0.51 ns
Philippines	13.01**	0.60 ns	−0.12*	0.03 ns	54.5*	4.9 ns	−30.0 ns	22.5 ns	0.21 ns
South Korea	13.92 ns	0.79 ns	−0.05 ns	0.89 ns	68.6*	17.9 ns	241.3 ns	250.1 ns	0.52 ns
Illapel	14.41 ns	−0.80 ns	−0.04 ns	1.89 ns	52.0 ns	31.9 ns	200.7 ns	256.2 ns	0.37 ns
Chepica	12.44*	−00.34 ns	−0.07**	0.66 ns	50.1 ns	16.4 ns	29.7 ns	75.2 ns	0.25 ns
Osorno	19.1*	0.49 ns	−0.07 ns	1.10 ns	100.7 ns	17.0 ns	54.8 ns	160.7 ns	0.68 ns

The levels of significance (ns, non-significant; *significant at 5%; **significant at 1%; using the F-test) are indicated.

**Figure 1:**
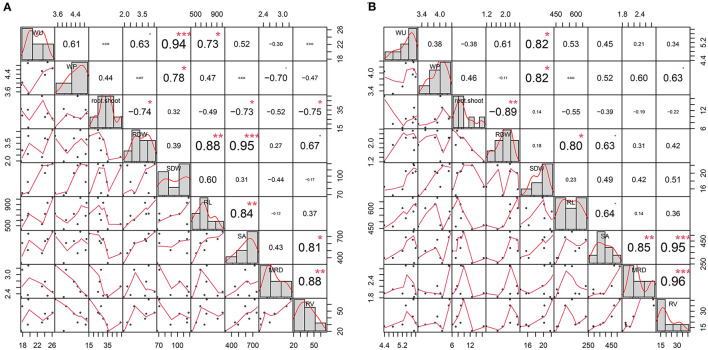
Plot representing phenotypic distribution and correlation for water consumption (WU), water productivity (WP), root and shoot biomass (RDW and SDW, respectively), and root traits (RL, Root length; MRD, Mean Root Diameter; RV, Root Volume; SA, Surface Area), evaluated under well-watered **(A)** and water-deficit **(B)** conditions. The diagonal line of the plot shows the histograms and the distribution of the observed phenotype values. The lower off-diagonal is the scatterplot between the traits, whereas the upper off-diagonal represents the correlation value between traits. *, **, and *** denote the level of significance at 5, 1, and 0.1%, respectively.

The Pearson correlation coefficients among WP, root and leaves biomass, and root traits are presented in Figure 1. Under the WW condition, WP showed a positive and significant (*p* < 0.01) correlation with SDW (0.78). Similarly, WU was significantly and positively associated with SDW (0.94) and RL (0.73). Furthermore, RDW showed strong and significant positive correlations with RL (0.88), SA (0.95), and RV (0.67). In particular, WP had a negative correlation with MRD (−0.70) (Figure 1A). These results indicate that, under the WW condition, greater shoot biomass production and a reduction in MRD are associated with more efficient use of water in the plant. On the other hand, under the WD condition, WP was significantly (and positively) correlated with SDW (0.82) and positively correlated with MRD (0.6) and RV (0.63), although not significant (*p* = 0.11 and 0.09, respectively). Furthermore, RDW showed a positive correlation with RL (0.80) and SA (0.63) (Figure 1B). These results indicate that, under WD conditions, WP is strongly associated with greater shoot biomass production.

### Response of Root Traits to Drought Stress

**Figure 2:**
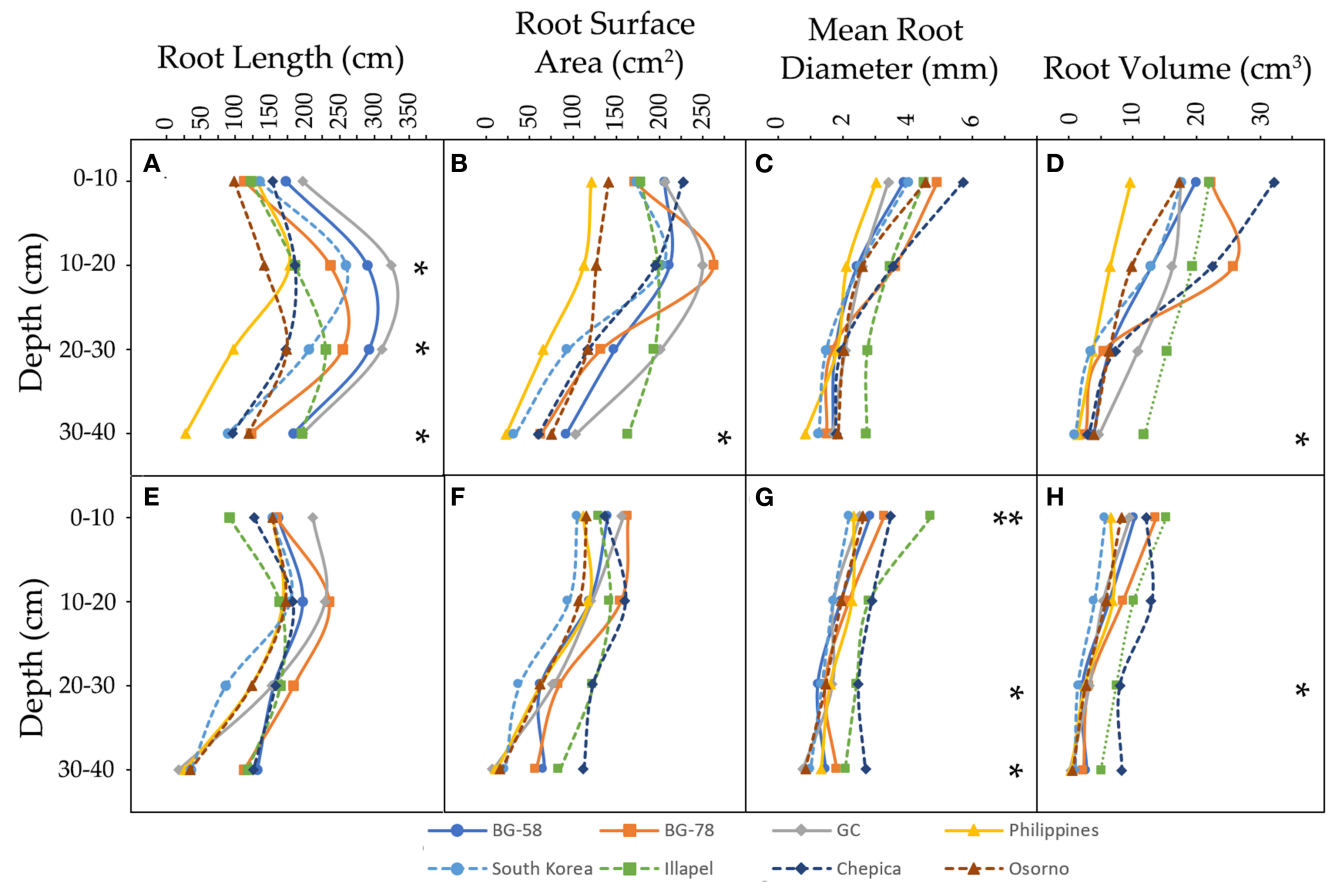
Distribution of root traits [root length **(A,E)**, root surface area **(B,F)**, mean root diameter **(C,G)**, and root volume **(D,H)**] in four depth layers (0–10, 10–20, 20–30, and 30–40 cm) of eight bottle gourd genotypes, grown under well-watered **(A–D)** and water-deficit conditions **(E–H)**. * and ** denote the level of significance at 5 and 1% respectively.

Figure 2 shows the four root traits evaluated at four different soil depth layers (0–10, 10–20, 20–30, and 30–40 cm). The maximum root depth was similar among the eight genotypes in both water regimes. However, the water regimes changed the root trait values through the different layers of depth. In all layers under the WD condition, the traits decreased by at least 50% compared with the WW condition. Notably, in all genotypes in the WD treatment, the RL presented the highest value in the second depth layer (10–20 cm), while the SA, MRD, and RV traits were decreased as the depth increased in almost all genotypes (except for the Philippines and Chepica genotypes, which had the highest values in the second depth layer; Supplementary Table 3).

Under the WW condition, significant genotypic differences in RL values were detected for most substrate layers (except 0–10 cm), while differences in SA and RV only were observed in the deepest layer (30–40 cm) (Figure 2 and Supplementary Table 3). In this sense, the GC and Philippines genotypes showed the highest and lowest values of RL, respectively, both being significantly different from each other. Similarly, the Philippines genotype presented the lowest SA and RV values, while the Illapel genotype had significantly high values in both traits. On the other hand, in the WD treatment, three layers (0–10, 20–30, and 30–40 cm) presented significant genotypic differences with regard to MRD and only two substrate layers (20–30 and 30–40 cm) with regard to RV (Supplementary Table 3). In particular, the bottle gourd accessions from Illapel and Chepica showed the highest values in MRD and RV in all substrate layers, while the South Korea and GC genotypes had the lowest values in the 0–10 and 20–30 cm layers (Supplementary Table 3). In this regard, it should be noted that, under the WD condition, the Illapel and Chepica genotypes had the highest WP values and presented the highest MRD and RV values (Supplementary Table 2). Furthermore, these genotypes in the first depth layers showed low (or close to general average) RL and MRD values; however, as the substrate depth increases, all the root trait values in these genotypes also increase. This may indicate that the Illapel and Chepica genotypes tend to explore the deeper layers of the substrate, which could be related to a higher capture of water from the substrate under drought conditions.

The RL, SA, and RV root traits were classified into different classes based on root diameter intervals, i.e., 0–0.5, 0.5–1.0, 1.0–1.5, 1.5–2.0, 2.0–2.5, 2.5–3.0, 3.0–3.5, 3.5–4.0, and 4.0–4.5 mm. This was performed to compare the fine root distribution across different diameter intervals under different depths for both water regimes.

**Figure 3:**
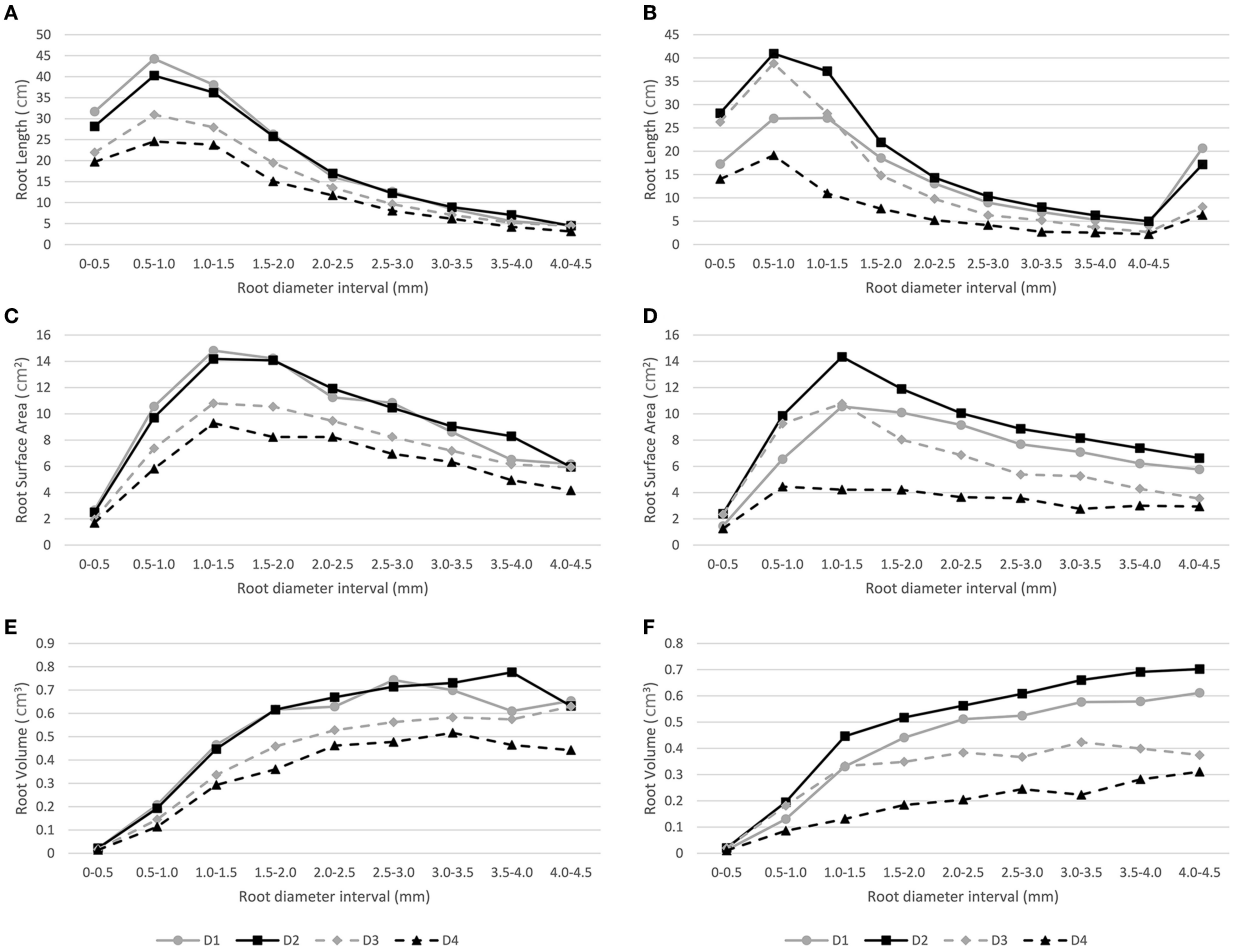
Distribution of root traits (i.e., RL, Root length; RV, Root Volume; SA, Surface Area) across nine root diameter intervals under well-watered (**A,C,E**, respectively) and water-deficit (**B,D,F**, respectively) conditions in four depth substrate layers (D1: 0–10 cm, D2: 10–20 cm, D3: 20–30 cm, D4: 30–40 cm).

The highest RL values in the four depths and both water conditions were recorded in the 0.5–1.0 mm diameter interval, then the values decrease according to the increase in diameter intervals. Moreover, in the 0.5–10 mm interval, significant genotypic differences were detected for the second depth layer (10–20 cm) under the WD condition, with the GC and Illapel genotypes having the highest and lowest values, respectively. Similarly, for SA, the highest values were observed in the 1.0–1.5 mm interval, and then, as the diameter of the intervals increased, the SA values decreased. It should be noted that under the WD regime, the fourth depth layer (30–40 cm) showed stable SA values after a diameter interval of 0.5–1.0 mm. Moreover, the RV values increased according to the increase in root diameter intervals independent of water regime and depth layer (Figure 3).

### Principal Component Analysis

**Figure 4:**
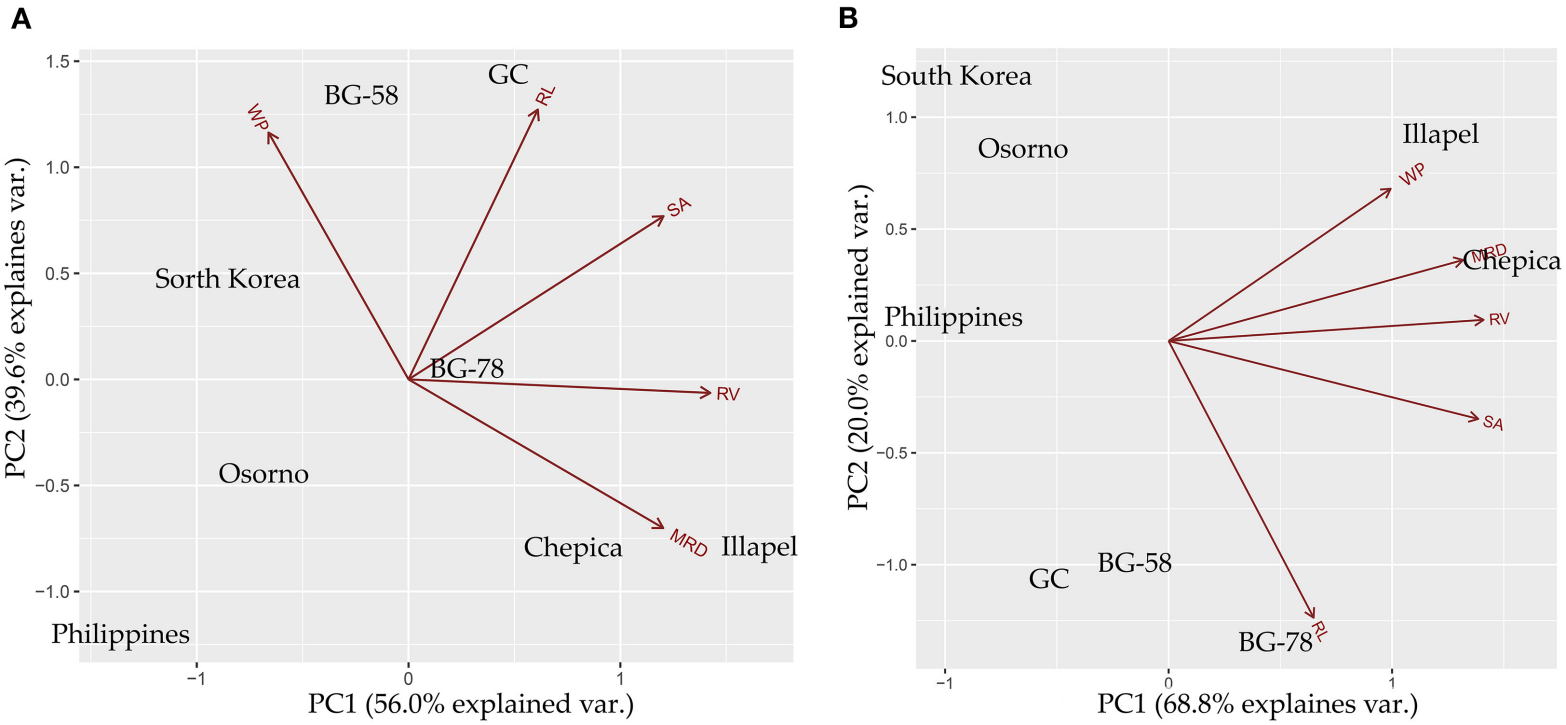
Principal components analysis (PCA) biplot for bottle gourd genotypes based on water productivity (WP) and root traits (RL, Root length; MRD, Mean Root Diameter; RV, Root Volume; SA, Surface Area), evaluated under well-watered **(A)** and water-deficit **(B)** conditions.

A principal component analysis was carried out to discover the most contributing root traits under two contrasting water regimes and for the differentiation of drought-tolerant and sensitive bottle gourd genotypes. Under the WW condition, the two first principal components (PCs) explained 96% of the cumulative variance observed, with 56% at the first and 40% at the second axes, with eigenvalues of 1.67 and 1.40, respectively (Figure 4). The parameters that contributed to positive loading to the first principal component (PCA1) were RL, SA, MRD, and RV; while for the second component (PCA2), the parameters were WP, RL, and SA. In contrast, the WP contributed with negative loading to the first component, while MRD and RV contributed with negative loading to the second component. Under the WD condition, the first two PCs explained 89% (69 and 20%, respectively). PC1 was composed only of positive loadings (WP, RL, SA, MRD, and RV). In contrast, PC2 consisted of RL and SA as negative loadings and WP, RV, and MRD as positive loadings.

Figure 4A (WW condition) shows that MRD has an opposite direction to WP, which is consistent with the negative correlation observed between these two traits. In this sense, the BG-58 and GC genotypes were grouped with high values of WP and low MRD values. On the other hand, the Illapel and Chepica genotypes had high MRD values and low WP values. Under the WD condition (Figure 4B), BG-58 and GC were grouped with low values of WP, while the Illapel and Chepica genotypes were differentiated by high values of MRD, RV, and WP, which is consistent with the positive correlation observed between these three traits. This result suggests that two of the Chilean genotypes (Chepica and Illapel) adopted a strategy of drought tolerance associated with MRD and RV growth, which was more efficient for WP compared with other genotypes.

### WP, Biomass Production, and Root Traits Analyzed by the Relative Distance Plasticity Index (RDPI)

Plasticity responses to a water deficit were compared for WP, root traits, and biomass production by means of the RDPI. In this sense, the Chilean genotypes had significantly higher plasticity in WP (Table 3), with Osorno genotype being significantly different from the Asiatic and South African genotypes. Similarly, the Chilean genotypes had the highest RDPI values in RDW; however, these values were low (<0.5). In particular, the Illapel and Chepica bottle gourds showed the highest RDPI values for MRD when compared with the other six genotypes. This indicates that the Chilean genotypes presented a higher phenotypic plasticity response under drought conditions (Table 3). In On the contrary, with regard to SDW, the highest RDPI values were observed in the South African plants, with the values for BG-58 and GC being significantly higher than those for the Illapel and Chepica genotypes. Regarding root traits, only the plasticity of RL showed a significant difference among the genotypes, for which the BG-58, GC, and South Korea genotypes were significantly higher than the Illapel and Chepica genotypes.

**Table 3:** Relative distance plasticity index (RDPI) considering water productivity (WP), root and shoot biomass (RDW and SDW, respectively) and root traits (RL, Root length; MRD, Mean Root Diameter; RV, Root Volume; SA, Surface Area), assessed in eight bottle gourd genotypes subjected to a water-deficit condition.

**Genotypes**	**WP**	**root:shoot**	**RDW**	**SDW**	**RL**	**SA**	**RV**	**MRD**
BG-58	0.13b	0.54ab	0.26a	0.70a	0.21ab	0.28a	0.35a	0.12a
BG-78	0.09b	0.53ab	0.20a	0.66ab	0.13bc	0.18a	0.27a	0.13a
GC	0.13b	0.61ab	0.28a	0.76a	0.25a	0.36a	0.44a	0.14a
Philippines	0.12b	0.66a	0.15a	0.64ab	0.07cd	0.19a	0.31a	0.14a
South Korea	0.10b	0.46b	0.20a	0.61abc	0.21ab	0.32a	0.43a	0.13a
Illapel	0.19ab	0.26c	0.29a	0.45c	0.17b	0.23a	0.40a	0.20a
Chepica	0.15b	0.48b	0.26a	0.49bc	0.04d	0.22a	0.40a	0.21a
Osorno	0.27a	0.52ab	0.34a	0.63ab	0.18ab	0.31a	0.43a	0.18a

*G, genotype; genotype means followed by different letters were significantly different (p ≤ 0.01) using Fisher's LSD test. Means with different letters within a column are significantly different at p <0.01 according to the Fisher's LSD test*.

## Discussion

Root architectural phenotypes play primary roles in the capture of water and soil resources and are important for maintaining crop yield under water-stress conditions. According to Lynch (2019), specific root phenotypes or root ideotypes are promising breeding targets for more stress-resilient and resource-efficient crops. Based on this idea, this study examined the root morphology of eight *L. siceraria* genotypes and investigated the various root traits at different substrate depths and root diameter intervals under a water-deficit condition. For instance, different root-related traits, such as primary root length, number and length of lateral roots, and average root diameter, have been proposed as important traits that contribute to regulating water uptake, which is critical under drought stress (Gorim and Vandenberg, 2017; Mwenye et al., 2018; Brunel-Saldias et al., 2020; Gao et al., 2020). In this study, a statistically significant effect of water deficit was reported over all the evaluated root traits. Furthermore, all root traits decreased in response to drought; however, the decrease rate of MRD, SA, and RV was smaller in the genotypes with higher WP (i.e., Illapel and Chepica). Thus, we suggest that bottle gourd genotypes with higher MRD, SA, and RV are more efficient in the use of water. In this sense, the Illapel and Chepica genotypes increased the MRD, SA and RV at the expense of the RL, while the South African genotypes (i.e., BG-58, BG-78, and GC) increased the RL and reduced the MRD and RV. These genotypic differences in terms of root length, diameter, and surface area may suggest that they have an important role in *L. siceraria* in response to water deficit. Plants with a higher main root diameter have more growth potential as there is a direct relation with water absorption (Richards et al., 2001; Zhou et al., 2018). In addition, it has been reported that root diameter controls the root surface area and length, hence encapsulating the overall effect in terms of root length per dry biomass allocated to the root system (Turner et al., 2001).

On the other hand, the total root length, which is important in the acquisition of water and soluble elements, is reduced due to water deficit. However, in this study, the South African genotypes showed the highest root length in fine (<2 mm diameter) and very fine roots ( ≤0.5 mm diameter) in the second (10–20 cm) and third (20–30 cm) layers, while the Illapel and Chepica genotypes presented the highest root length in the thicker roots (>2 mm diameter) in the last layer (30–40 cm) (Supplementary Data Sheet S1). It has been stated that plants with root systems with smaller root diameter and long fine roots are more suited to drought environments (Khadka et al., 2020). There is evidence that in different crops under water-deficit conditions, the drought-resistant genotypes showed higher fine root length than the susceptible genotype (i.e., soybean—Chun et al., 2021; wheat—Henry et al., 2012; lentil—Gorim and Vandenberg, 2017). Moreover, a genotype with a higher total root length in the middle or deeper layers of the soil may be resistant to drought due to an efficient distribution of roots (Hamedani et al., 2020). In this context, our results may indicate that *L. siceraria* genotypes use different mechanisms of adaptation to tolerate water deficits. This could imply that the South African genotypes are able to exploit water resources more efficiently in the second and third soil layers, as one of the main functions of fine roots is water uptake, while the Illapel and Chepica genotypes, have thicker roots with higher proportions of RV and SA gives them the advantage of being able to penetrate deeper into soil layers, and they are therefore more efficient at transporting water from long distances.

The size and shape of pots are generally regarded as a limitation in the root growth and water uptake, a process sometimes termed “pot binding” or “root binding” (Sinclair et al., 2017). In this study, the size of the pot can be a limitation to the root growth, however, Poorter et al. (2012), who conducted a metanalysis study, concluded that the plant biomass per unit rooting volume is more relevant than pot size per se, being recommended avoid plant biomass to pot volume ratio's values larger than 2 gL^−1^ (and preferably <1 gL^−1^) if want to minimize growth restrictions for harvest. In this context, this study reported ratios of biomass/root volume between 1.06 and 2.58 gL^−1^ for each genotype treatment, which is in agreement with the proposed by Poorter et al. (2012) and more recently by Turner, 2018. In addition, the duration of our experiment was 4 weeks, which suggests that the plants may grow presumably well in all pot sizes because as revised by Poorter et al. (2012), after 4 weeks of growth, the biomass is reduced in the smallest volume pots (Turner, 2018).

This study also evaluated whether root trait relationships with shoot biomass are affected by drought. In this sense, the water-deficit condition caused a reduction in plant growth, which was expressed as a reduction in all plant traits, except for the root:shoot ratio. The increase in the root:shoot ratio was a result of a greater reduction in aboveground biomass rather than an increase in root biomass because the absolute root dry weight under the water-deficit condition was not greater than that under the well-watered condition. In addition, this ratio is often observed to increase under adverse conditions such as drought (Liu et al., 2004; Xu et al., 2015). The reason for the increased root-to-shoot ratio could be due to the limited supply of water; hence, root growth occurs at the expense of the shoot. According to Lozano et al. (2020), the stronger impact of drought on leaves than on root traits may be linked to differences in the physiology of roots and leaves. The plant root system is the organ responsible for the uptake of water and is the first responder to many types of stress (Brunner et al., 2015; Weemstra et al., 2016; Lozano et al., 2020). Moreover, due to the ability of roots to grow toward wetter patches in the soil, the effects of water shortage can be minimized (Eapen et al., 2005; Lozano et al., 2020). Furthermore, from a genetic point of view, the root:shoot ratio also showed genotypic variation in bottle gourd in response to a water deficit. A significant genotypic variation in the root:shoot ratio in response to a water deficit has also been reported in other crop species (i.e., rice—Cui et al., 2008; sesame—Hamedani et al., 2020; chickpea—Ramamoorthy et al., 2017). These findings suggest that a higher root:shoot ratio in response to drought stress is an advantage that enables plants to cope with water-limited conditions, depending on the genotype, the degree, and duration of drought stress, or the synergistic/antagonistic interaction of these factors (Xu et al., 2015).

Plasticity is critical to plant acclimation and survival under environmental stress, especially drought (Pierik and Testerink, 2014). In fact, the plasticity of a root system under drought conditions is considered to be a key trait driving the modification of the growth and structure of roots in response to a water deficit (Bristiel et al., 2019). Our results indicated that all traits studied showed some level of plasticity in response to a water deficit, even though the plasticity presented here was relatively low (RDPI < 0.5), with the exception of the root:shoot dry weight ratio and the shoot dry weight. Stotz et al. (2021) pointed out that the costs of phenotypic plasticity tend to increase in plants under stressful conditions, resulting in lower phenotypic plasticity values. Moreover, these costs and limits can be different among traits, with morphological traits being more limited under stressful conditions due to higher costs, resulting in lower phenotypic plasticity. Our results supported the theory of Stotz et al. (2021), since the WP and root traits showed relatively low RDPI values, with low statistical differences among genotypes. Despite these results, in our previous study, Zacarias Rafael et al. (2020) evaluated the plasticity of the morphological and physiological traits of the roots in bottle gourd under water-deficit conditions, revealing that the Illapel genotype had the highest plasticity index for WP. Similarly, in our previous study, the Illapel genotype, along with the Chepica and Osorno genotypes, showed greater RDPI values in MRD and WP. Notwithstanding these results, according to Schneider et al. (2020a), to better understand and interpret plasticity, first, it is necessary to have a comprehensive understanding of the utility of individual phenes (i.e., root phenes). In fact, previous studies have demonstrated that phenotypic plasticity is phene-specific, not necessarily genotype-specific (Schneider et al., 2020a,b). Thus, further research is necessary for evaluating whether plasticity in root diameter growth may be related to WP performance in genotypes of bottle gourd under water-deficit conditions.

Understanding the associations among the relevant root traits associated with drought adaptation/tolerance is important for improving the selection of superior genotypes for drought tolerance and the breeding of bottle gourd. In this context, PCA is a powerful statistical procedure that reduces the dimensions of the variables and, in our case, helps to discriminate highly correlated traits in order to identify clusters of individuals in response to drought stress (Bahrami et al., 2014). Moreover, PCA biplot analysis has been effectively used by other researchers for screening drought-tolerant bottle gourd (Mashilo et al., 2017, 2018; Zacarias Rafael et al., 2020). In this study, PCA analysis identified groups of genotypes with different strategies to face (or tolerate) drought stress based on WP and root traits (i.e., MRD, RL, SA, and RV). In this sense, the Illapel and Chepica genotypes were grouped together based on high values for WP, MRD, and RV, and were separated from the Asiatic, Osorno, and two South African genotypes (GC and BG-58). Similar findings were observed by Zacarias Rafael et al. (2020), who showed that the Illapel and Chepica genotypes were grouped together based on water-use efficiency in WUEwp (whole plant), WUEi (intrinsic), and WUEins (instantaneous), while the South African genotypes were localized in a different cluster. In addition, Mashilo et al. (2017), who performed a biplot analysis based on the physiological parameters in *L. siceraria* under drought stress, suggested that BG-58, BG-78, and GC are drought-tolerant genotypes based on high values of physiological traits (gs, stomatal conductance; A, net CO_2_ assimilation rate). In the present study, under water-deficit conditions, the Chilean and South African bottle gourds showed different strategies for tolerating drought stress. In particular, water-deficit conditions may lead to an increase in the diameter and volume of roots, which, in turn, may contribute to improving water capture under these conditions.

The need for drought-resistant crops is critical and will surely grow in the coming years, considering the notable degradation of soil and water resources, and the accelerating effects of global climate change. In this context, the *L. siceraria* breeding programs for rootstock should therefore incorporate key root phenotypes (including morphological and anatomical traits) that focus on water capture and nutrient uptake, and which are suited for sustainable production in cucurbit crops (Suárez-Hernández et al., 2017, 2022), as well as those of watermelon, melon and cucumber (Suárez-Hernández et al., 2017, 2022). On the other hand, the traits that affect the metabolic efficiency of root growth and soil exploration should therefore also be important components of drought tolerance and plant productivity, the effects of which should be evaluated in different combinations of rootstock varieties.

## Conclusion

The evaluated bottle gourd showed genotypic variation for relevant root traits under water-deficit conditions. The Illapel and Chepica genotypes were associated with better WP, MRD, RV, and SA to different soil depths, while BG-58 and BG-78 increased the TRL in fine and very fine roots (≤0.5 and <2 mm diameter, respectively) and reduced the MRD and RV. Thus, both groups of bottle gourd used different strategies associated with root trait modifications in response to a water deficit. Notably, the genotypic variation observed for these root traits at different substrate depths, especially under water-deficit conditions, was also associated with drought tolerance in *L. siceraria*. Therefore, the variation in root traits at different substrate depths should also be considered in the selection and breeding programs of bottle gourds.

## Data Availability Statement

The original contributions presented in the study are included in the article/Supplementary Material, further inquiries can be directed to the corresponding author/s.

## Author Contributions

RC-S, DZ, CM, and FM-P conceived the research plans. CM, FM-P, and RC-S analyzed the phenomic data, wrote the first draft of the manuscript, and reviewed and edited the final version of the manuscript. RC-S, DZ, CM, and LD supervised the field experiments. RC-S and CM performed the data curation. All authors reviewed and approved the paper for publication.

## Funding

This work was supported by FONDECYT (Grant Number 11180278).

## Conflict of Interest

The authors declare that the research was conducted in the absence of any commercial or financial relationships that could be construed as a potential conflict of interest.

## Publisher's Note

All claims expressed in this article are solely those of the authors and do not necessarily represent those of their affiliated organizations, or those of the publisher, the editors and the reviewers. Any product that may be evaluated in this article, or claim that may be made by its manufacturer, is not guaranteed or endorsed by the publisher.

## References

[B1] Aslam W. Noor R. S. Hussain F. Ameen M. Ullah S. Chen H. (2020). Evaluating morphological growth, yield, and postharvest fruit quality of cucumber (*Cucumis sativus* L.) grafted on cucurbitaceous rootstocks. Agriculture 10, 101–120. 10.3390/agriculture10040101

[B2] Awad W. Byrne P. F. Reid S. D. Comas L. H. Haley S. D. (2018). Great Plains winter wheat varies for root length and diameter under drought stress. Agron. J. 110, 226–235. 10.2134/agronj2017.07.0377

[B3] Bahrami F. Arzani A. Karimi V. (2014). Evaluation of yield-based drought tolerance indices for screening safflower genotypes. Agron. J. 106, 1219–1224. 10.2134/agronj13.0387

[B4] Bristiel P. Roumet C. Violle C. Volaire F. (2019). Coping with drought: root trait variability within the perennial grass *Dactylis glomerata* captures a trade-off between dehydration avoidance and dehydration tolerance. Plant Soil 434, 327–342. 10.1007/s11104-018-3854-8

[B5] Brunel-Saldias N. Ferrio J. P. Elazab A. Orellana M. Del Pozo A. (2020). Root architecture and functional traits of spring wheat under contrasting water regimes. Front. Plant Sci. 11, 581140. 10.3389/fpls.2020.581140 33262777 PMC7686047

[B6] Brunner I. Herzog C. Dawes M. A. Arend M. Sperisen C. (2015). How tree roots respond to drought. Front. Plant Sci. 6, 547. 10.3389/fpls.2015.00547 26284083 PMC4518277

[B7] Chun H. C. Sanghun L. E. E. Choi Y. D. Gong D. H. Jung K. Y. (2021). Effects of drought stress on root morphology and spatial distribution of soybean and adzuki bean. J. Integr. Agric. 20, 2639–2651. 10.1016/S2095-3119(20)63560-2

[B8] Comas L. H. Becker S. R. Cruz V. M. V. Byrne P. F. Dierig D. A. (2013). Root traits contributing to plant productivity under drought. Front. Plant Sci. 4, 442. 10.3389/fpls.2013.00442 24204374 PMC3817922

[B9] Cui K. Huang J. Xing Y. Yu S. Xu C. Peng S. (2008). Mapping QTLs for seedling characteristics under different water supply conditions in rice (*Oryza sativa*). Physiol. Plant. 132, 53–68. 10.1111/j.1399-3054.2007.00991.x 18251870

[B10] Decker-Walters D. S. Wilkins-Ellert M. Chung S. M. Staub J. E. (2004). Discovery and genetic assessment of wild bottle gourd [*Lagenaria siceraria* (Mol.) Standley; Cucurbitaceae] from Zimbabwe. Econ. Bot. 58, 501–508. 10.1663/0013-0001(2004)0580501:DAGAOW2.0.CO;2

[B11] Eapen D. Barroso M. L. Ponce G. Campos M. E. Cassab G. I. (2005). Hydrotropism: root growth responses to water. Trends Plant Sci. 10, 44–50. 10.1016/j.tplants.2004.11.004 15642523

[B12] Ellsworth P. Z. Feldman M. J. Baxter I. Cousins A. B. (2020). A genetic link between leaf carbon isotope composition and whole-plant water use efficiency in the C4 grass Setaria. Plant J. 102, 1234–1248. 10.1111/tpj.14696 31968138

[B13] Fahad S. Bajwa A. A. Nazir U. Anjum S. A. Farooq A. Zohaib A.. (2017). Crop production under drought and heat stress: plant responses and management options. Front. Plant Sci. 8, 1147. 10.3389/fpls.2017.01147 28706531 PMC5489704

[B14] Fahad S. Saud S. Yajun C. Chao W. Depeng W. (eds.). (2021f). Abiotic Stress in Plants. London: IntechOpen. 10.5772/intechopen.91549

[B15] Fahad S. Sönmez O. Saud S. Wang D. Wu C. Adnan M.. (eds.). (2021a). “Plant growth regulators for climate-smart agriculture,” in Footprints of Climate Variability on Plant Diversity (Boca Raton, FL: CRC Press). 80–98. 10.1201/9781003109013

[B16] Fahad S. Sonmez O. Saud S. Wang D. Wu C. Adnan M.. (eds.). (2021b). “Climate change and plants: biodiversity, growth and interactions,” in Footprints of Climate Variability on Plant Diversity (Boca Raton, FL: CRC Press). 1–6. 10.1201/9781003108931

[B17] Fahad S. Sonmez O. Saud S. Wang D. Wu C. Adnan M.. (eds.). (2021c). “Developing climate resilient crops: improving global food security and safety,” in Footprints of Climate Variability on Plant Diversity (Boca Raton, FL: CRC Press). 82–108. 10.1201/9781003109037

[B18] Fahad S. Sönmez O. Saud S. Wang D. Wu C. Adnan M.. (eds.). (2021e). “Engineering tolerance in crop plants against abiotic stress,” in Footprints of Climate Variability on Plant Diversity (Boca Raton, FL: CRC Press). 158–174. 10.1201/9781003160717

[B19] Fahad S. Sönmez O. Turan V. Adnan M. Saud S. Wu C.. (eds.). (2021d). “Sustainable soil and land management and climate change,” in Footprints of Climate Variability on Plant Diversity (Boca Raton, FL: CRC Press). 1–15. 10.1201/9781003108894

[B20] Gao X. B. Guo C. Li F. M. Li M. He J. (2020). High soybean yield and drought adaptation being associated with canopy architecture, water uptake, and root traits. Agronomy 10, 608. 10.3390/agronomy10040608

[B21] Gorim L. Y. Vandenberg A. (2017). Root traits, nodulation and root distribution in soil for five wild lentil species and *Lens culinaris* (Medik.) grown under well-watered conditions. Front. Plant Sci. 8, 1632. 10.3389/fpls.2017.01632 28993782 PMC5622593

[B22] Hamedani N. G. Gholamhoseini M. Bazrafshan F. Amiri B. Habibzadeh F. (2020). Variability of root traits in sesame genotypes under different irrigation regimes. Rhizosphere 13, 100190. 10.1016/j.rhisph.2020.100190

[B23] Henry A. Cal A. Batoto T. C. Torres R. O. Serraj R. (2012). Root attributes affecting water uptake of rice (*Oryza sativa*) under drought. J. Exp. Bot. 63, 4751–4763. 10.1093/jxb/ers150 22791828 PMC3427995

[B24] Iqbal S. Wang X. Mubeen I. Kamran M. Kanwal I. Díaz G. A.. (2022). Phytohormones trigger drought tolerance in crop plants: outlook and future perspectives. Front. Plant Sci. 12, 799318. 10.3389/fpls.2021.799318 35095971 PMC8792739

[B25] Kamran M. Malik Z. Parveen A. Zong Y. Abbasi G. H. Rafiq M. T.. (2019). Biochar alleviates Cd phytotoxicity by minimizing bioavailability and oxidative stress in pak choi (*Brassica chinensis* L.) cultivated in Cd-polluted soil. J. Env. Manag. 250, 109500. 10.1016/j.jenvman.2019.109500 31513996

[B26] Khadka K. Earl H. J. Raizada M. N. Navabi A. A. (2020). Physio-morphological trait-based approach for breeding drought tolerant wheat. Front. Plant Sci. 11, 715. 10.3389/fpls.2020.00715 32582249 PMC7286286

[B27] Kistler L. Montenegro A. Smith B. D. Grifford J. A. Green R. E. Newsom L. A.. (2014). Transoceanic drift and the domestication of African bottle gourds in the Americas. Proc. Nat. Acad. Sci. U.S.A. 111, 2397–2941. 10.1073/pnas.1318678111 24516122 PMC3939861

[B28] Kobiakova J. A.. (1930). The bottle gourd. Bull. Appl. Bot. Genet. Plant. Breed. 23, 475–520.

[B29] Liu H. S. Li F. M. Xu H. (2004). Deficiency of water can enhance root respiration rate of drought-sensitive but not drought-tolerant spring wheat. Agric. Water Manage. 64, 41–48. 10.1016/S0378-3774(03)00143-4

[B30] Lozano Y. M. Aguilar-Trigueros C. A. Flaig I. C. Rillig M. C. (2020). Root trait responses to drought are more heterogeneous than leaf trait responses. Funct. Ecol. 34, 2224–2235. 10.1111/1365-2435.13656

[B31] Lynch J.. (2018). Rightsizing root phenotypes for drought resistance. J. Exp. Bot., 69, 3279–3292. 10.1093/jxb/ery048 29471525

[B32] Lynch J.. (2019). Root phenotypes for improved nutrient capture: an underexploited opportunity for global agriculture. N. Phytol. 223, 548–564. 10.1111/nph.15738 30746704

[B33] Marchiori P. E. R. Machado E. C. Sales C. R. G. Espinoza-Núñez E. Magalhães Filho J. R. Souza G. M.. (2017). Physiological plasticity is important for maintaining sugarcane growth under water deficit. Front. Plant Sci. 8, 2148. 10.3389/fpls.2017.02148 29326744 PMC5742411

[B34] Mashilo J. Odindo A. O. Shimelis H. A. Musenge P. Tesfay S. Z. Magwaza L. S. (2017). Drought tolerance of selected bottle gourd [*Lagenaria siceraria* (Molina) Standl.] landraces assessed by leaf gas exchange and photosynthetic efficiency. Plant Physiol. Biochem. 120, 75–87. 10.1016/j.plaphy.2017.09.022 28988036

[B35] Mashilo J. Odindo A. O. Shimelis H. A. Musenge P. Tesfay S. Z. Magwaza L. S. (2018). Photosynthetic response of bottle gourd [*Lagenaria siceraria* (Molina) Standl.] to drought stress: relationship between cucurbitacins accumulation and drought tolerance. Sci. Hortic. 231, 133–143. 10.1016/j.scienta.2017.12.027

[B36] Mwenye O. J. Van Rensburg L. Van Biljon A. Van der Merwe R. (2018). “Seedling shoot and root growth responses among soybean (Glycine max) genotypes to drought stress,” in Soybean—Biomass, Yield and Productivity, ed, Kasai M. (London, UK: IntechOpen), 10.

[B37] Opazo I. Toro G. Salvatierra A. Pastenes C. Pimentel P. (2020). Rootstocks modulate the physiology and growth responses to water deficit and long-term recovery in grafted stone fruit trees. Agric. Water Manage. 228, 105897. 10.1016/j.agwat.2019.105897

[B38] Payus C. Huey L. A. Adnan F. Rimba A. B. Mohan G. Chapagain S. K.. (2020). Impact of extreme drought climate on water security in North Borneo: case study of Sabah. Water 12, 1135. 10.3390/w12041135

[B39] Peterson B. G. Carl P. Boudt K. Bennett R. Ulrich J. Zivot E.. (2018). Package 'PerformanceAnalytics'. Vienna: R Team Cooperation.

[B40] Pierik R. Testerink C. (2014). The art of being flexible: how to escape from shade, salt, and drought. Plant Physiol. 166, 5–22. 10.1104/pp.114.239160 24972713 PMC4149730

[B41] Poorter H. B?hler J. van Dusscholten D. Climent J. Postma J. A. (2012). Pot size matters: a meta-analysis of the effects of rooting volume on plant growth. Funct. Plant Biol. 39, 839–850 10.1071/FP12049 32480834

[B42] R Core Development Team (2020). R: A Language and Environment for Statistical Computing. Vienna: R Foundation for Statistical Computing.

[B43] Ramamoorthy P. Lakshmanan K. Upadhyaya H. D. Vadez V. Varshney R. K. (2017). Root traits confer grain yield advantages under terminal drought in chickpea (*Cicer arietinum* L.). Field Crops Res. 201, 146–161. 10.1016/j.fcr.2016.11.004 28163361 PMC5221670

[B44] Richards R. A. Condon A. G. Rebetzke G. J. (2001). “Traits to improve yield in dry environments,” in Application of Physiology in Wheat Breeding, eds M. P. Reynolds, J. I. Ortiz-Monasterio, and A. McNab (Mexico: CIMMYT), 88–100.

[B45] Schlumbaum A. Vandorpe P. (2012). A short history of *Lagenaria siceraria* (bottle gourd) in the Roman provinces: morphotypes and archaeogenetics. Veg. Hist. Archaeobot. 21, 499–509. 10.1007/s00334-011-0343-x

[B46] Schneider H. Klein S. Hanlon M. Brown K. Kaeppler S. Lynch J. (2020a). Genetic control of root anatomical plasticity in maize. Plant Genome 13, e20003. 10.1002/tpg2.20003 33016634 PMC12807228

[B47] Schneider H. Klein S. Hanlon M. Nord E. Kaeppler S. Brown K.. (2020b). Genetic control of root architectural plasticity in maize. J. Exp. Bot. 71, 3185–97. 10.1093/jxb/eraa084 32080722 PMC7260711

[B48] Sinclair T. R. Manandhar A. Shekoofa A. Rosas-Anderson P. Bagherzadi L. Schoppach R.. (2017). Pot binding as a variable confounding plant phenotype: theoretical derivation and experimental observations. Planta 245, 729–735. 10.1007/s00425-016-2641-0 27999989

[B49] Sithole N. Modi A. T. (2015). Responses of selected bottle gourd [*Lagenaria siceraria* (Molina Standly)] landraces to water stress. Acta Agric. Scand. Sect. B Soil Plant Sci. 65, 350–356. 10.1080/09064710.2015.1012109

[B50] Stotz G. C. Salgado-Luarte C. Escobedo V. M. Valladares F. Gianoli E. (2021). Global trends in phenotypic plasticity of plants. Ecol. Lett., 24, 2267–2281. 10.1111/ele.13827 34216183

[B51] Strock C. F. Burridge J. Massas A. S. Beaver J. Beebe S. Camilo S. A.. (2019). Seedling root architecture and its relationship with seed yield across diverse environments in Phaseolus vulgaris. Field Crops Res. 237, 53–64. 10.1016/j.fcr.2019.04.012

[B52] Suárez-Hernández A. M. Grimaldo-Juárez O. Ceceña-Durán C. Vázquez-Angulo J. C. Carrazco-Peña L. D. Avendaño-Reyes L.. (2022). Influence of seed and fruit characteristics of *Lagenaria siceraria* on production and quality of grafted watermelon. Horticulturae 8, 242. 10.3390/horticulturae8030242

[B53] Suárez-Hernández A. M. Grimaldo-Juárez O. García-López A. M. González-Mendoza D. Huitrón-Ramírez M. V. (2017). Evaluación de portainjertos criollos de *Lagenaria siceraria* en la producción de sandía injertada. Idesia 35, 39–44. 10.4067/S0718-34292017005000002

[B54] Turner N. C.. (2018). Imposing and maintaining soil water deficits in drought studies in pots. Plant Soil 439, 45–55. 10.1007/s11104-018-3893-1

[B55] Turner N. C. Wright G. C. Siddique K. H. M. (2001). Adaptation of grain legumes (pulses) to water-limited environments. Adv. Agron. 71, 193–231. 10.1016/S0065-2113(01)71015-2

[B56] Ulas A. Doganci E. Ulas F. Yetisir H. (2019). Root-growth characteristics contributing to genotypic variation in nitrogen efficiency of bottle gourd and rootstock potential for watermelon. Plants 8, 77. 10.3390/plants8030077 30934590 PMC6473247

[B57] Vu V. Q.. (2011). ggbiplot: A ggplot2 based biplot. R Package, version 0.55. Available online at: https://github.com/vqv/ggbiplot

[B58] Wasaya A. Zhang X. Fang Q. Yan Z. (2018). Root phenotyping for drought tolerance: a review. Agron. J. 8, 1–19. 10.3390/agronomy8110241

[B59] Weemstra M. Mommer L. Visser E. J. Ruijven J. Kuyper T. W. Mohren G. M.. (2016). Towards a multidimensional root trait framework: a tree root review. N. Phytol. 211, 1159–1169. 10.1111/nph.14003 27174359

[B60] Xu W. Cui K. Xu A. Nie L. Huang J. Peng S. (2015). Drought stress condition increases root to shoot ratio via alteration of carbohydrate partitioning and enzymatic activity in rice seedlings. Acta Physiol. Plant. 37, 9. 10.1007/s11738-014-1760-0

[B61] Yavuz D. Seymen M. Süheri S. Yavuz N. Türkmen Ö. Kurtar E. S. (2020). How do rootstocks of citron watermelon (*Citrullus lanatus* var. citroides) affect the yield and quality of watermelon under deficit irrigation? Agric. Water Manage. 241, 106351. 10.1016/j.agwat.2020.106351

[B62] Yetisir H. Sari N. Yucel S. (2003). Rootstock resistance to fusarium wilt and effect on watermelon fruit yield and quality. Phytoparasitica 31, 163–169. 10.1007/BF02980786

[B63] Yetisir H. Uygur V. (2009). Plant growth and mineral element content of different gourd species and watermelon under salinity stress. Turk. J. Agric. Forest 33, 65–77 10.1080/01904160903470372

[B64] Zacarias Rafael D. Arriagada O. Toro G. Mashilo J. Mora-Poblete F. Contreras-Soto R. I. (2020). Plasticity of the root system architecture and leaf gas exchange parameters are important for maintaining bottle gourd responses under water deficit. Plants 9, 1697. 10.3390/plants9121697 33287101 PMC7761539

[B65] Zhou G. Zhou X. Nie Y. Bai S. H. Zhou L. Shao J.. (2018). Drought-induced changes in root biomass largely result from altered root morphological traits: evidence from a synthesis of global field trials. Plant Cell Environ. 41 2589–2599. 10.1111/pce.13356 29879755

