# Supplementary material for: Variation in Root-Related Traits Is Associated With Water Uptake in Lagenaria siceraria Genotypes Under Water-Deficit Conditions
Source: Front Plant Sci. 2022 Jun 2;13:897256. doi: 10.3389/fpls.2022.897256 (PMC9201500; doi:10.3389/fpls.2022.897256)
Supplement: Supplementary file 2 [file Table_1.docx]

Supplementary Material

# Supplementary Tables

**Table Supplementary S1**. List of the eight-bottle gourd used in the study with description of collection sites: origin, region, location, and geographical coordinates.

| **Code** | **Country of origin** | **Province/Region** | **Location** | **Lat S** | **Long E** |
| --- | --- | --- | --- | --- | --- |
| BG-58 | South Africa | Limpopo | Kgohloane | 23°47’39.76’’ | 29°22’13.45’’ |
| BG-78 | South Africa | Limpopo | Moletjie-Mabokelele | 23°45’14.69’’ | 29°17’36.6’’ |
| GC | South Africa | KwnZulu-Natal | La Lucia | 29°45’19.98’’ | 31°04’33.41’’ |
| Philippines | Philippines L. siceraria var. hispida | No information | No information | No information | No information |
| South Korea | South Korea L. siceraria var. hispida | No information | No information | No information | No information |
| Illapel | Chile | Coquimbo | Illapel | 31°35’29.58’’ | 70°45’67’’ |
| Chépica | Chile | Chépica | O’Higgins | 35°26’42.00’’ | 72°01’28.40’’ |
| Osorno | Chile | Osorno | Loncoche | 39°22’31.44’’ | 72°36’28.69’’ |

| **Genotype** | **Treatment** | **WU** | **WP** | **RSDWR** | **RDW** | **SDW** | **RL** | **RSA** | **MRD** | **RV** |
| --- | --- | --- | --- | --- | --- | --- | --- | --- | --- | --- |
| **ANOVA** | |  |  |  |  |  |  |  |  |  |
| **SV** | **Treatment** | **Significance** | | | | | | | | |
| G | T1 | ns | ns | * | ns | ns | *** | ns | ns | ns |
| G | T2 | ns | ns | ns | *** | ns | ns | ns | * | ns |
| BG-58 | T1 | 25.04 a | 4.94 a | 31.35 b | 3.82 a | 117.09 a | 930.77 ab | 664.08 a | 2.34 a | 38.76 a |
| BG-78 | T1 | 24.36 a | 4.37 a | 27.50 bc | 3.91 a | 104.88 a | 721.85 bc | 634.91 a | 2.78 a | 46.12 a |
| GC | T1 | 26.39 a | 4.66 a | 25.86 bc | 4.62 a | 119.68 a | 1025.00 a | 767.00 a | 2.46 a | 47.64 a |
| Philippines | T1 | 17.83 a | 3.91 a | 36.19 ab | 1.96 a | 68.84 a | 434.07 d | 324.97 a | 2.30 a | 20.73 a |
| South Korea | T1 | 19.69 a | 4.90 a | 35.30 ab | 2.59 a | 90.56 a | 685.61 ab | 507.64 a | 2.36 a | 29.91 a |
| Illapel | T1 | 19.81 a | 3.52 a | 16.24 c | 4.12 a | 72.53 a | 726.89 c | 739.01 a | 3.28 a | 67.22 a |
| Chepica | T1 | 18.18 a | 4.02 a | 29.10 bc | 2.90 a | 72.46 a | 606.65 bc | 606.49 a | 3.15 a | 57.18 a |
| Osorno | T1 | 23.54 a | 4.54 a | 49.92a | 2.32 a | 117.20 a | 528.40 a | 463.26 a | 2.63 a | 33.24 a |
| BG-58 | T2 | 5.84 a | 3.87 a | 9.13 a | 2.24 ab | 20.43 a | 631.47 a | 388.67 a | 1.94 b | 19.38 |
| BG-78 | T2 | 5.71 a | 3.95 a | 8.00 a | 2.53 a | 20.04 a | 679.95 a | 461.21 a | 2.21 b | 25.09 a |
| GC | T2 | 5.25 a | 3.55 a | 6.36 a | 2.56 a | 15.89 a | 602.48 a | 362.87 a | 1.95 b | 17.75 a |
| Philippines | T2 | 4.82 a | 3.31 a | 7.54 a | 1.92 b | 14.36 a | 464.07 a | 302.47 a | 2.09 b | 15.80 a |
| South Korea | T2 | 5.77 a | 4.11 a | 12.96 a | 1.70 bc | 21.99 a | 444.33 a | 257.56 a | 1.83 b | 11.93 a |
| Illapel | T2 | 5.40 a | 4.33 a | 9.28 a | 2.23 ab | 20.53 a | 526.16 a | 482.83 a | 2.91 a | 35.28 a |
| Chepica | T2 | 5.74 a | 4.36 a | 9.92 a | 2.23 ab | 22.36 a | 576.98 a | 531.28 a | 2.89 a | 40.76 a |
| Osorno | T2 | 4.43 a | 4.05 a | 16.82 a | 1.22 c | 16.51 a | 473.57 a | 302.55 a | 1.96 b | 16.21 a |

**Table Supplementary S2**. Analysis of variance for the genotypic effect on water productivity (WP), root and leaves biomass (RSDWR, RDW and SDW) and root traits (RL, MRD, RV and RSA) evaluated in eight bottles gourd genotypes under well-watered (WW) and water deficit (WD) conditions.

SV: source of variation; G: Genotype; T1: well-watered; T2: water-deficit; Genotype means followed by different letter were significantly different (P <= 0.01) by Fisher's LSD test. The levels of significance (ns non-significant; * significant at 5%; ** significant at 1%; *** significant at 0.1% by the F-test) are indicated.

**Table Supplementary S3**. Analysis of variance for the genotypic effect to root traits (RL, RV, MRD and RSA) in four depth layers (0-10, 10-20, 20-30 and 30-40 cm) of eight bottle gourd genotypes, grown under well-water and well-water regimes.

| **G** | **T** | **D1** | | | | **D2** | | | | **D3** | | | | **D4** | | | |
| --- | --- | --- | --- | --- | --- | --- | --- | --- | --- | --- | --- | --- | --- | --- | --- | --- | --- |
|  |  | *RL* | *RSA* | *MRD* | *RV* | *RL* | *RSA* | *MRD* | *RV* | *RL* | *RSA* | *MRD* | *RV* | *RL* | *RSA* | *MRD* | *RV* |
| **ANOVA** | |  |  |  |  |  |  |  |  |  |  |  |  |  |  |  |  |
| **SV** | **T** | **Significance** | | | | | | | | | | | | | | | |
| G | T1 | ns | ns | ns | ns | * | ns | ns | ns | * | ns | ns | ns | * | * | ns | * |
| G | T2 | ns | ns | ** | ns | ns | ns | ns | ns | ns | ns | * | * | ns | ns | * | * |
| BG-58 | T1 | 172.5 a | 206.31a | 3.8 a | 19.9 a | 290.0 a | 212.1 a | 2.5 a | 13.0 a | 292.6 ab | 148.4 a | 1.8 a | 6.4 a | 184.0 a | 92.3 abc | 1.7 a | 3.9 b |
| BG-78 | T1 | 113.1 a | 172.4 a | 4.9 a | 22.4 a | 236.5 abc | 263.6 a | 3.6 a | 25.9 a | 254.6 ab | 132.2 a | 1.7 a | 5.6 a | 122.8 ab | 62.6 bc | 1.5 a | 2.6 b |
| GC | T1 | 197.3 a | 206.3 a | 3.4 a | 17.5 a | 324.9 a | 249.9 a | 2.6 a | 16.3 a | 310.8 a | 201.6 a | 2.1 a | 10.9 a | 197.5 a | 104.1 ab | 1.7 a | 4.8 b |
| Philippines | T1 | 131.9 a | 121.4 a | 3.0 a | 9.7 a | 178.0 bc | 112.7 a | 2.1 a | 6.5 a | 97.1 c | 66.5 a | 1.8 a | 3.8 a | 28.7 b | 22.2 c | 0.8 a | 1.4 b |
| South Korea | T1 | 135.2 a | 173.1 a | 4.0 a | 17.7 a | 259.3 ab | 204.8 a | 2.5 a | 12.9 a | 206.2 abc | 93.9 a | 1.5 a | 3.4 a | 89.3 ab | 32.9 bc | 1.3 a | 1.0 b |
| Illapel | T1 | 122.9 a | 178.5 a | 4.5 a | 22.1 a | 185.5 bc | 198.1 a | 3.5 a | 19.4 a | 229.8 ab | 194.3 a | 2.8 a | 15.4 a | 195.5 a | 164.1 ab | 2.7 a | 12.0 a |
| Chepica | T1 | 153.2 a | 227.2 a | 5.7 a | 32.2 a | 186.2 bc | 195.4 a | 3.5 a | 22.5 a | 172.1 bc | 117.7 a | 2.0 a | 7.4 a | 95.6 ab | 60.2 bc | 1.8 a | 3.1 b |
| Osorno | T1 | 98.4 a | 141.5 a | 4.6 a | 17.4 a | 141.6 c | 127.2 a | 2.6 a | 9.9 a | 173.9 bc | 116.9 a | 2.0 a | 6.3 a | 119.0 ab | 75.4 bc | 1.9 a | 3.9 b |
| BG-58 | T2 | 159.3 a | 139.7 a | 2.8 bc | 10.3 a | 194.3 a | 118.7 a | 1.9 a | 6.3 a | 153.1 a | 61.6 a | 1.4 b | 2.0 c | 129.5 a | 66.3 a | 1.5 bc | 2.8 b |
| BG-78 | T2 | 157.2 a | 163.3 a | 3.3 bc | 13.7 a | 232.6 a | 155.0 a | 2.2 a | 8.4 a | 181.6 a | 83.0 a | 1.6 b | 3.2 bc | 110.4 a | 56.6 a | 1.8 abc | 2.4 b |
| GC | T2 | 209.4 a | 157.3 a | 2.5 bc | 9.6 a | 227.1 a | 121.2 a | 1.7 a | 5.4 a | 150.7 a | 76.1 a | 1.7 b | 3.2 bc | 16.0 a | 6.2 a | 0.8 c | 0.2 b |
| Philippines | T2 | 152.4 a | 111.5 a | 2.4 bc | 6.5 a | 166.7 a | 117.8 a | 2.3 a | 6.9 a | 121.1 a | 61.0 a | 1.6 b | 2.5 c | 25.2 a | 10.6 a | 1.3 bc | 0.4 b |
| South Korea | T2 | 151.7 a | 104.0 a | 2.2 c | 5.7 a | 177.2 a | 94.3 a | 1.7 a | 4.0 a | 83.9 a | 37.1 a | 1.4 b | 1.4 c | 33.5 a | 20.8 a | 1.0 bc | 1.0 b |
| Illapel | T2 | 89.6 a | 130.1 a | 4.7 a | 15.3 a | 161.7 a | 143.0 a | 2.8 a | 10.2 a | 162.6 a | 123.3 a | 2.4 a | 7.4 ab | 115.7 a | 84.0 a | 2.1 ab | 5.0 b |
| Chepica | T2 | 124.6 a | 137.3 a | 3.5 b | 12.1 a | 180.5 a | 159.7 a | 2.9 a | 13.0 a | 155.6 a | 121.8 a | 2.5 a | 8.0 a | 123.5 a | 111.7 a | 2.7 a | 8.3 a |
| Osorno | T2 | 151.3 a | 115.3 a | 2.6 bc | 8.2 a | 170.3 a | 106.9 a | 1.9 a | 5.8 a | 121.6 a | 62.4 a | 1.5 b | 2.6 c | 32.5 a | 16.1 a | 0.8 c | 0.7 b |

SV: source of variation; G: Genotype; T: Treatment; T1: well-watered; T2: water-deficit; Genotype means followed by different letter were significantly different (P <= 0.01) by Fisher's LSD test. The levels of significance (ns non-significant; * significant at 5%; ** significant at 1%; *** significant at 0.1% by the F-test) are indicated.
